# Supplementary material for: High-quality and easy-to-regenerate personal filter
Source: PLoS One. 2022 Jun 8;17(6):e0268542. doi: 10.1371/journal.pone.0268542 (PMC9176855; doi:10.1371/journal.pone.0268542)
Supplement: Attachment — Submitted filename: Response to Reviewer.docx [file pone.0268542.s002.docx]

# Authors’ responses

(5.4.2022)

Reviewer #1: Fraenkl et al. discuss the potential of wearing a mask connected with a backpack made of easily washable cloth for improved filtration efficiencies. The ideas that increasing the number of filtration layers and decreasing face velocity of air through the mask material can lead to improved filtration efficiency are well known. In this case, the authors take advantage of these principles by connecting a facemask to a large surface area backpack made with polyester fleece fabric, which can be easily washed. As a result, exchange of air during breaths is accomplished through the surface area of the backpack. The idea is simple but innovative and appears more effective than I would have imagined. The degree of filtration (99.9%) is surprising but perhaps can be explained by the extremely slow face velocities and long path length for the particles. While there are some challenging aspects of this design with respect to implementation as compared to typical filtering facepiece respirators, I believe that the innovation can be beneficial in resource-limited settings. I have listed specific comments below:

Above all we would like to thank you for studying our article, for your questions and suggestions. We have included some of your suggestions in the article and we believe that you have helped us to improve the comprehensibility and contribution of our article. The following are the answers to your questions and an explanation of some of our views.

**Question 1: Figure 2, panel B: How many times was the experiment repeated? Are there graphs with error bars showing the variation in performance with multiple tests?**

Authors' response: Thanks for your question. I realized that there is no direct link between Figure 2A-C and the experimental data including error bars. So I added a link to the supplementary Figure S6 to the manuscript. I also added information that the measurement was repeated 4 times.

**Question 2. Figure 2, panel B: There is a stark increase in filtration efficiency when the number of layers were changed from 2 to 4 for mask areas 150 cm2 and 1200 cm2, which is unusual. Can the authors explain this behavior?**

Authors' response: Thanks for your question. This is our explanation added into the article: “At the bottom of Figure 2B, variations in penetration value are seen in both the perpendicular and vertical directions. Variations gradually disappear with increasing number of layers. It can be assumed that the PES fleece fabric is partially inhomogeneous and with a larger number of layers, its properties in the direction of air flow are averaged.”

**Question 3. Page 12, line 149: “Additionally, the number of penetrating particles decreases exponentially with an increasing number of filter layers…”
Can the authors relate their observations regarding exponential decrease in captured particles and increase in filtration efficiency with theoretical predictions and what other reports have observed?
e.g. Zangmeister, Christopher D., et al. "Filtration efficiencies of nanoscale aerosol by cloth mask materials used to slow the spread of SARS-CoV-2." ACS nano 14.7 (2020): 9188-9200.**

Authors' response:

We would like to show here the theoretical assumptions, our results in the form of graphs and the results from the article ACS nano. We compared them with a simple calculation.

The clipping below from the above-mentioned article (ACS nano 14.7 (2020): 9188-9200) shows realition between filtration effciency *FE* and thickness of filter *L*.

Relation between filtration eficiency *FE* and penetration *P* is such

$$FE=1-P$$

Than particle penetration decreases exponentially with increasing filter thickness L.

$$P=e^{-\gamma L}$$

The same formulation can be found in Aerosol Technology form William C. Hinds where *γ* represent coefficient of fractional penetration and *L* represents filter thickness.

**Our measurements** showing the exponential decrease of the particles with increasing thickness (number of layers) of the filter can be found in Figure 2B. These values are plotted and interpolated with an exponential function in the figure below.

The data are unfortunately scattered for the reason given in the answer to question no.2. For example, the variance of ±10% of data can also be found in the ACS nano 14.7 (2020): 9188-9200.

In the article ACS nano 14.7 (2020): 9188-9200 is mentioned that Filtering efficiency is increasing monotonically with number of cloth layers.

I extracted data from figure 3D below and…

… plotted them together with a simple theoretical calculation (red circles) based on filtration efficiency of single layer 15% as subtraced from figure 3D. Both curves have a similiar trend until the layer 6-7 is reached.

A simple calculation example is shown below:

From the above, I assume that our results and the results reported in ACS nano (14.7 (2020): 9188-9200) are consistent with the theoretical assumption that “*the number of penetrating particles decreases exponentially with an increasing number of filter thickness”* (number of layers).

**Question 4. Figure 3: Please label the subpanels and different objects in the images. What actions are being performed? The image needs to have detailed information for the readers to understand the process better.**

Authors' response: Thanks for the suggestion. I added a labels and improved the captions accordingly.

**Question 5. Is it possible for the authors to show more experimental results in figure 4 as compared to the infographic table presented currently? e.g. Fig S8 provides valuable information about pressure drop in a single component. Multiple components can be plotted together on the same graph, including total pressure drop measured from the combined setup. Important experimental results can be moved from the Supplementary to the main manuscript for benefit of readers.**

Authors' response:

We would prefere not add further figures concerning the pressure drop of the filter kit or its individual parts to the manuscript. We believe that the main news is that it is possible to assemble a filter cartridge with high efficiency from a commonly available easily regenerable material. That the pressure drop of the cartridge itself is very low, i.e. about a quarter of the pressure drop of the filter kit. We know that the pressure drop of the set will always strongly depend on the parts actually used to assemble the kit, i.e. the choice of flexible hoses, their diameter, bending, type of mask. Therefore, we prefer to provide this information with the attached information or discuss it on request.
